# Positive Regulation of Decidualization by l-Type Amino Acid Transporter 1 (lat1) in Pregnant Mice

**DOI:** 10.3390/nu8110704

**Published:** 2016-11-05

**Authors:** Xiaojie Wang, Dongmei Tan, Jing Ma, Hao Liang, Qian Zhang, Yi Tan, Jiang Wang, Wenping Luo

**Affiliations:** 1 Laboratory Animal Center, Chongqing Medical University, Chongqing 400016, China; duomiaicha@163.com (X.W.); dongmei_tan@126.com (D.T.); mj5658700@126.com (J.M.); lianghaowd@163.com (H.L.); jenniferzhang85@126.com (Q.Z.); tanyee66@hotmail.com (Y.T.); 2 Chong Qing Obstetrics and Gynecology Hospital, Chongqing 400013, China; leidoug@gmail.com; 3 Chongqing Key Laboratory of Oral Diseases and Biomedical Sciences, Chongqing 401147, China; 4 Stomatological Hospital of Chongqing Medical University, Chongqing 401147, China; 5 Chongqing Municipal Key Laboratory of Oral Biomedical Engineering of Higher Education, Chongqing 401147, China

**Keywords:** decidualization, l-amino acid transporter-1 (lat1), stromal cell, pregnant mice

## Abstract

Amino acids have an important role in the pre and post implantation of placenta and embryo development. l-type amino-acid transporter 1 (lat1) is responsible for the transportation of large neutral amino acids and is mainly expressed in human fetal liver, placenta, brain, etc. This study is the first to investigate the expression of lat1 in the early pregnancy of mouse uteri and its role in the process of decidualization. Endometrial stromal cells of a mouse model were used to evaluate decidualization from Day 4–8 of pregnancy in vitro followed by lat1 knock down by small interfering RNA and by a competitive inhibitor of Leucine transport 2-aminobicyclo-(2,2,1)-heptane-2-carboxylic acid (BCH). The effects of lat1 on decidualization in vivo were assessed by injecting BCH into the uterine horns. The mRNA and protein expressions of lat1 in the implantation sites were higher than that in the inter-implantation sites and were localized in the luminal and gland epithelium, stromal and decidual cells. Its increased expression (*p* < 0.05) was associated with artificial decidualization as well as activation of prl expression. Down-regulation of lat1 expression in these cells by siRNA and BCH inhibited the decidual progression in vitro. Inhibition of lat1 transportation by BCH controlled decidual progression in vivo also accompanied the down-regulation of prl, lat1 expression in the decidual area and embryo size on Day 8 of pregnancy. In conclusion, these results revealed that lat1 might play an important role in the decidual progression both in vitro and in vivo.

## 1. Introduction

Amino acids play an important role in the pre and post implantation of placenta and embryo development. Some essential amino acids such as l-arginine have influence on the embryogenesis, embryonic/fetal survival, and growth in both humans and rodents. Dietary Arginine supplementation during early pregnancy in rats enhances embryo implantation through stimulation of PI3K/PKB/mTOR/NO signaling pathway [1]. Amino acids such as Leucine and Arginine stimulate the mammalian target of rapamycin (mTOR) activity during the blastocyst stage, and similarly help in the trophoblast outgrowth by in vitro and in vivo implantation [2]. Two amino acid transport systems were associated with mouse-ovulated oocytes or with preimplantation of mouse embryos: (1) sodium independent L-transport system; and (2) sodium dependent A-transport system [3,4,5]. l-type amino-acid transporter 1 (lat1) is a sodium-independent amino acid transporter and is a member of l-type transport system, which is responsible for transporting large neutral amino acids and is expressed in normal endothelial cells such as human fetal liver, placenta, brain, bone marrow, spleen, testis and some tumors [6]. Few studies have demonstrated the requirement of active transport of amino acids for successful implantation and placentation [7]. In addition, during the process of placentation, lat1 might play a role in the invasive phenotype of trophoblast giant cells [2]. Embryo implantation and decidualization are critical for the establishment of successful pregnancy. Decidualization is characterized by proliferation and differentiation of endometrial stromal cells (ESCs), followed by blastocyst attachment within 22–24 h on Day 4 (D4) of pregnancy [8,9,10]. Maternal decidua play a multi-functional role in the embryo–uterine dialog such as feto-maternal immuno-tolerance and placental development [11,12,13]. Although numerous molecules of the signaling pathway have been identified such as epidermal growth factor (EGF) and Hoxa10, which are necessary for the decidual development, but no evidence suggests the role of lat1 in the coordination of ovarian hormone with the embryo–uterine dialog in decidualization [8,12]. Our previous study indicated that lat1 was expressed in the mouse uterus during the early phase of placentation and promoted the outgrowth of ectoplacental cones (EPCs), which suggested that lat1 might participate in the early establishment of the placenta [14]. Hence, in the present study, we showed a strict-space-time and cell-specific expression of lat1 within the implantation sites in the maternal compartment. Furthermore, the study has examined the function of lat1 as a stimulant in the decidualization progression through prolactin (prl) in vitro and in vivo.

## 2. Experimental Section

### 2.1. Animals and Treatments

Adult female Kun-Ming mice, aged 6–8 weeks and weighing 20–25 g were purchased from Chongqing Medical University (Certificate No.: SCXK (YU) 20070001), and raised under a constant photoperiod (12 h light:12 h darkness). All animal procedures were approved by the Ethics Committee of Chongqing Medical University. Guidelines for the Care and Use of laboratory Animals in Research were followed. Virgin female mice were mated with fertile males of the same strain to induce pregnancy. Presence of a vaginal plug (next day morning) confirmed mating and was designated Day 1 of pregnancy (D1). Pregnant mice were injected with trypan blue by tail vein (Sigma, St. Louis, MO, USA, 4 mg/mL) to identify the implantation and inter-implantation sites and then were sacrificed from D4 to Day 8 (D8) of pregnancy everyday between 9:00 and 10:00 a.m. and tissues were collected for the subsequent experiments.

To observe the effects of lat1 on uterine decidualization and prolactin (prl) expression, mice were randomly divided into four groups on D4 of pregnancy. Experimental groups were injected with: (1) inhibitor of lat1 activity, 2-aminobicyclo-(2,2,1)-heptane-2-carboxylic acid (BCH) (Sigma, St. Louis, MO, USA, 50 μg/mL, 5 µL); (2) inhibitor of lat1 activity, 2-aminobicyclo-(2,2,1)-heptane-2-carboxylic acid (BCH) (Sigma, St. Louis, MO, USA, 5 μg/mL, 5 µL); (3) NH_4_OH was employed as a solvent (Sigma, St. Louis, MO, USA, 1 mol/L, 5 µL) through two uterine horns (the junction of fallopian tubes and uterus); and (4) blank control group was without any treatment. Mice were sacrificed on D8 between 9:00 and 10:00 a.m. and tissues were collected for the subsequent experiments. Quantitative analyses of regional decidual development were according to the previously described method [15]. There were 15 mice sacrificed in each group.

### 2.2. Primary Culture of Uterine Endometrium ESC and Induction of Decidualization In Vitro Extraction and Induction of Decidualization

Uterine endometrial stromal cells (ESC) were isolated and cultured according to the previously described method [10]. There were 40 mice sacrificed in this experiment. In brief, on Day 4 of pregnancy, the lumen of the uterine horns were cleansed from fat tissue, then were placed in 30 IU Dispase (Invitrogen, Thermo Fisher Scientific, Waltham, MA, USA) and 0.25% trypsin (Invitrogen, Thermo Fisher Scientific, Waltham, MA, USA) for 1 h at 4 °C followed by 1 h at room temperature. The remaining tissues were incubated in phosphate buffer saline (PBS, Gibco, Carlsbad City, California, CA, USA) containing 0.5 mg/mL collagenase Type V (Invitrogen, Thermo Fisher Scientific, Waltham, MA, USA) at 37 °C for 30 min. Cells were plated at 4 × 10^5^ cells per 50 square centimeter in dishes that contained phenol red-free Dulbecco’s Modified Eagle’s Medium (DMEM) and Ham’s F-12 nutrient mixture (1:1) with 10% charcoal-stripped serum and antibiotic in a humidified incubator at 37 °C with 5% CO_2_. After an initial incubation for 1–2 h, the medium was removed from free-floating cells, and the cells that adhered to the culture dishes were transferred to the fresh medium (DMEM–F-12, 1:1) containing 10% charcoal-stripped fetal bovine serum (FBS, Gibco, Carlsbad, California, CA, USA), E2 (estradiol-17, 10 nM, Sigma, St. Louis, MO, USA), and P4 (progesterone, 1 μM, Sigma, St. Louis, MO, USA). This point of time was designated as 0 h and then the detection time to analyze was selected at 24 h interval after treatment. Examination of cytokeratin by immunostaining revealed the absence of epithelial cells at 24 h after treatment and, therefore, the expression of prl was chosen as a biochemical marker of decidualization in ESCs that were cultured up to 72 h.

#### 2.2.1. Addition of Inhibitor

ESCs extracted by the above described method were plated at 4 × 10^5^ cells per 50 square centimeter in the dishes and were randomly assigned to the following treatment groups for 48 h when decidualization was induced at 24 h (Group 1: Blank control without any treatment; Group 2: 0.05 μM BCH; Group 3: 0.1 μM BCH; Group 4: 0.5 μM BCH; Group 5: 2 μM BCH; and Group 6: 4 μM BCH), and then were cultured up to 72 h.

#### 2.2.2. Transfection

*Lat1*-targeting shRNAs were synthesized by Genechem (PCONGV102000025, Shanghai, China) and sequences were listed in Supplementary Materials Table S1. *Lat1* cDNA was purchased from Fulen Gene (H4509, Guangzhou, China). In order to overexpress lat1 exogenously, the cDNA sequence was sub-cloned into the pEGFP-N1 plasmid vector to harvest pEGFP-N1-*Lat1* plasmid.

Prior to decidualization of ESCs in vitro, shRNAs and pEGFP-N1-*Lat1* plasmid were transfected into the cultured ESCs at 60%–70% confluence according to the Lipofectamine^®^ 2000 Transfection Reagent protocol (11668019, Invitrogen, Thermo Fisher Scientific, Waltham, MA, USA). Briefly, 6 μL Lipofectamine^®^ 2000 was mixed with 3 μg shRNA or pEGFP-N1-*Lat1* plasmid to form complexes, and this mixture was then dispersed into a 6-well cell culture plate. The transfection efficiency with fluorescent-labeled shRNA of plasmid was observed by fluorescent microscope.

### 2.3. Immunocytochemistry

Mouse uteri from D4 to D8 of normal pregnancy and BCH treatment in vivo were fixed in 4% paraformaldehyde for 24 h, dehydrated and embedded in paraffin. Sections (4–6 μm) were then cut, deparaffinized and rehydrated. Endogenous peroxidase activity was blocked by incubating the sections in 3% peroxide in methanol for 20 min at room temperature. The sections were blocked nonspecifically by binding in 10% normal rabbit serum for 1 h at room temperature followed by incubation with rabbit anti-lat1 primary antibody (1:500, sc-134994, Santa Cruz Biotechnology, Dallas, TX, USA), rabbit anti-prl primary antibody (1:200, BA0601, Boster, Pleasanton, CA, USA), respectively for overnight at 4 °C. The sections were then subsequently incubated with horseradish peroxidase-conjugated goat anti-rabbit IgG (ZB-2301, Zhongshan Biotechnology, Beijing, China) for 40 min at room temperature. The secondary antibody was detected with 3,3′-diaminobenzidine solution (ZLI-9033, Zhongshan Biotechnology, Beijing, China). For some sections, primary antibody was replaced with normal rabbit IgG (2 ug/mL IgG instead of primary antibody) to serve as negative controls. Immunohistochemistry was performed on fifteen pregnant mice from each group and each sample was assayed three times. The positive expression was indicated by brown color in each region which was analyzed using the Image Pro-plus 6.0 software (Media Cybernetics, Rockville, MD, USA). The relative expression level was quantified using the IOD (integrated optical density), IOD = ∑(optical density × area).

### 2.4. Western Blot Analysis

Total protein samples from the tissues were isolated from Day 4 to 8 of pregnancy and total protein samples of cells were isolated from various treatment groups, separated on a 12% sodium dodecyl sulfate (SDS) polyacrylamide gel (20 μg protein per well), and were transferred on to a nitrocellulose membrane (Hybond™-C, Amersham Bio-Sciences, Piscataway, NJ, USA). The membranes were blocked with 5% non-fat milk in TRIS-buffered saline containing 0.1% Tween 20 at room temperature for 2 h and then were incubated at 4 °C overnight with the following primary antibodies: rabbit anti-lat1 primary antibody (1:1000, sc-134994, Santa Cruz Biotechnology), rabbit anti-gapdh (1:1000, 2118S, Cell Signaling Technology, Danvers, MA, USA) or rabbit anti-prl (1:200, BA14521, Boster, Wuhan, China). After incubation with the corresponding strain, secondary antibodies (HRP-labeled goat anti-rabbit IgG (H + L) (1:2000, A0208, Beyotime, Nantong, China) for 60 min at room temperature, the membranes were subjected to enhanced chemiluminescence (BeyoECL Plus, P0018, Beyotime, Shanghai, China). There were 15 mice sacrificed in each group and each sample was assayed three times.

### 2.5. Semiquantitative RT-PCR

Total RNA was isolated from decidualization of ESCs and mouse uteri using the TRIzol reagent (Invitrogen, Carlsbad City, California, CA, USA) according to the manufacturer’s instructions. Total RNA (2 μg) was reverse transcribed in 20 µL of reaction mixture containing 4 µL MgCl_2_, 25 mM; 2 µL Reverse Transcription 10× Buffer; 2 µL dNTP Mixture, 10 mM; 0.5 µL Recombinant RNasin^®^ Ribonuclease Inhibitor, 15 U AMV Reverse Transcriptase (High Conc.), and 0.5 µg random primers (A3500, Promega, Madison, WI, USA). The PCR was performed in a total volume of 25 µL containing 12.5 µL GoTaq^®^ Green Master Mix (M7122, Promega), 0.5 µM primers and 1 µL cDNA and was carried out for over 22 cycles in *β-Actin* which was employed as an internal control and 30 cycles for lat1. The thermal cycling conditions were as follows: 94 °C for 30 s, 55–59 °C for 30 s, and 72 °C for 30 s. The primers used in this study included *Lat1* Mus (NM_011404.3) (Forward: 5′-CTTTGTACAGCGGCCTCTTC-3′, Reverse: 5′-CAGGACATGACACCCAAGTG-3′) and *β-Actin* (Forward primer: 5′-AGCCATGTACGTAGCCATCC-3′, Reverse primer: 5′-CTCTCAGCTGTGGTGGTGAA-3′). There were 15 mice sacrificed per group and each sample was assayed three times.

### 2.6. Histochemistry

After fixation in 4% paraformaldehyde(PFA), serial sections of implantation sites (ISs) on D8 of pregnancy with 5 μm were deparaffinized, rehydrated in a graded series of alcohol and stained with hematoxylin–eosin (H&E). The areas of different regions from the ISs treated with BCH in vivo on D8 were quantified with ImageJ 1.43 software (National Institute of Health, Bethesda, MD, USA). The extent of different decidual regions were calculated in relation to the area of the whole decidua, designated as 100%. There were 15 mice sacrificed per group.

### 2.7. Statistical Analysis

Results were presented as mean ± SD (standard deviation). One-way analysis of variance followed by least-significant-difference test was used for statistical comparisons among multiple groups. For statistical comparisons between the two groups, an independent-sample *t*-test was used. Significant differences were assumed at *p* < 0.05 and highly significant differences were assumed at *p* < 0.01.

## 3. Results

### 3.1. Expression of Lat1 in Mouse Uteri during Implantation

**Figure 1:**
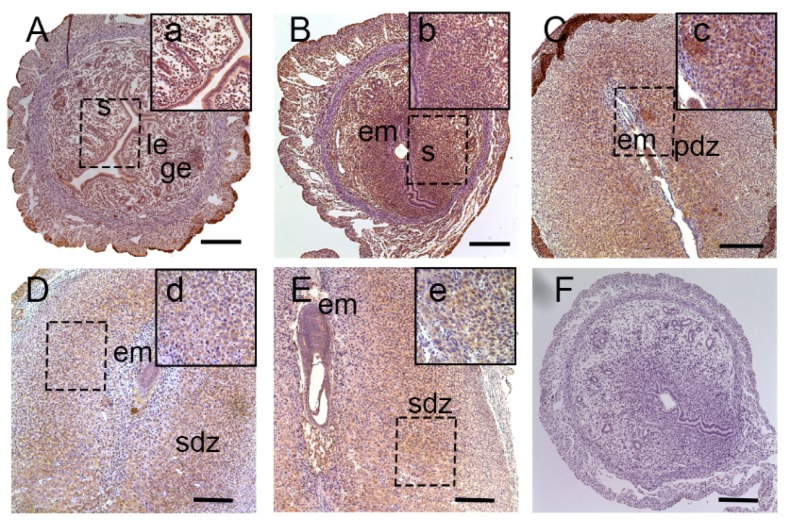
Immunostaining of lat1 in mouse uterus from D4 to D8 of pregnancy. Original magnification immunostainingof lat1 in mouse uterus from D4 to D8 of pregnancy on Days: 4 (**A**); 5 (**B**); 6 (**C**); 7 (**D**); and 8 (**E**) of pregnancy, 100 times magnification. Bar = 25 μm. a–e: inserts shows 400 times magnification; and (**F**): Negative control (IS: implantation sites, IIS: inter-implantation sites, em: embryo, ge: glandular epithelium, le: luminal epithelium, s: stromal, pdz: primary decidual zone, sdz: secondary decidual zone). There were 15 mice sacrificed on every day of pregnancy.

Pregnant mice on Day(D5) were injected with trypan blue by tail vein to identify the implantation sites (blue stained) and inter-implantation sites (Supplementary Materials Figure S1). To examine the spatiotemporal expression pattern of lat1 in mouse uteri from D4 to D8 of pregnancy, immunohistochemical staining was performed as shown in Figure 1. Lat1 was mainly distributed in the luminal and glandular epithelial cells, and ESC cytoplasm on D4 of pregnancy (Figure 1A). Increased expression was noted in the decidualizing stromal cells in the cytoplasm throughout the endometrium at the IS on D5 (Figure 1B). ISs on D6 (Figure 1C) of pregnancy showed lat1 expression in the cytoplasm of decidual cells in primary decidualization zone (pdz) and embyro (Figure 1C). On D7 (Figure 1D) and D8 (Figure 1E), lat1 was located in the decidual cells cytoplasm in the second decidualization zone cytoplasm (sdz) and embryo (Figure 1D,E). These results revealed that there were some link in the pattern of expression between lat1 and decidualization.

**Figure 2:**
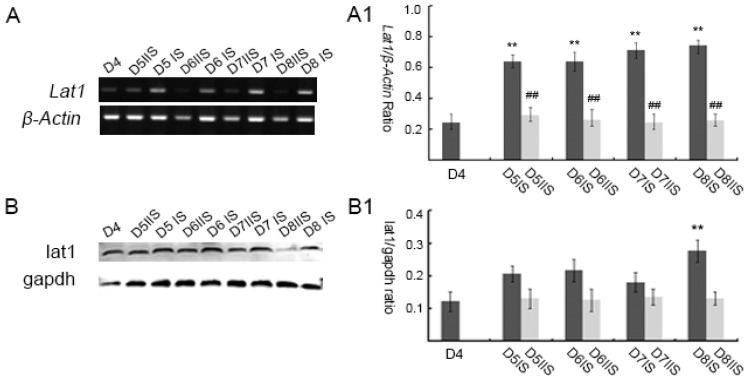
Expression of lat1 in mouse uterus from D4 to D8 of pregnancy. To distinguish implantation and inter-implantation sites, trypan blue was injected by caudal vein on D5 and D6: (**A**, **A1**) semi-quantitative RT-PCR analysis of the *Lat1* mRNA in mouse uterus from D4 to D8 of pregnancy; and (**B**, **B1**) Western blot analysis of the lat1 protein in mouse uterus from D4 to D8 of pregnancy (IS: implantation sites, IIS: inter-implantation sites). ** *p* < 0.01, as compared with D4; ^##^ *p* < 0.01, the expression of lat1 in inter-implantation sites compared with corresponding implantation sites. There were 15 mice sacrificed on every day of pregnancy.

Consistent with the above results, further quantitative analyses of lat1 were performed by Western blotting and semi-quantitative RT-PCR. Expression of *Lat1* mRNA in the mouse uteri from D4 to D8 of pregnancy was examined by semi-quantitative RT-PCR (Figure 2A, A1). *Lat1* mRNA was observed in the mouse uteri, but very low in the inter-implantation sites (IIS). Compared with D4 of pregnancy, the levels of lat1 mRNA were increased significantly on D5 to D8 of pregnancy IS, *p* < 0.05.

Similarly, lat1 proteins were present in mouse uteri from D4 to D8 of pregnancy. Western blot analyses revealed that the level of lat1 from D5 to D8 of pregnancy in IS was higher than that in corresponding IIS. The expression of lat1 protein was significantly increased on D8 compared to D4 of pregnancy, (Figure 2B, B1, *p* < 0.05). Although the levels of protein and mRNA did not change consistently from D5 to D7 in IS, both displayed similar variational tendencies and the peak levels appeared on D8 of pregnancy.

### 3.2. Up or Down Regulation Effect of lat1 on prl Expression in the Decidualization of ESCs

The evident spatiotemporal expression pattern of lat1 in mouse uteri from D4 to D8 of pregnancy implied a role in controlling decidualization of ESCs. Up to 72 h after P4 (progesterone) and E2 (estradiol-17) treatment, the prl protein level and cell morphology were analyzed to ensure successful induction of decidualization in vitro (Supplementary Materials Figures S2 and S3).

**Figure 3:**
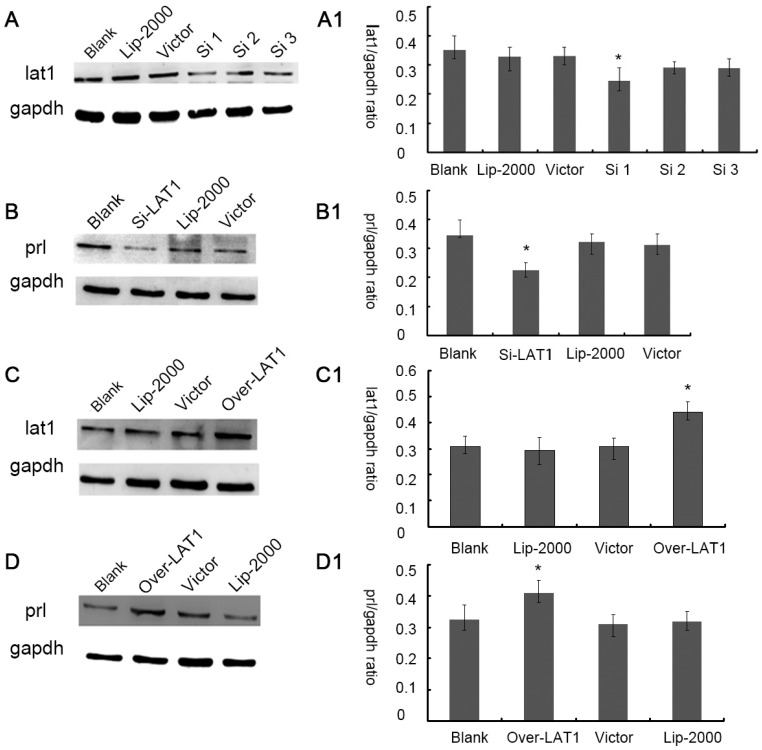
Regulations of lat1 Effect prl Expression in decidualization of ESC: (**A**, **A1**) Confirmation of lat1 lower expression using Western blot analysis. Three sequences ShRNA called Si1, Si2, and Si3 were used in transfecting combined with 2000 transfection reagent (lip2000) and verify the Si1 sequence can effectively silent lat1; (**B**, **B1**) Transfected with lat1shRNA, showing the lower expression of prl by Western blot analysis; (**C**, **C1**) Confirmation of lat1 over expression after transfecting with pEGFP-N1-*Lat1* plasmid (Over-lat1) using Western blot analysis; (**D**, **D1**) Transfected with pEGFP-N1-*Lat1* (Over-lat1) showing higher expression of prl by Western blot analysis. * *p* < 0.05, as compared with blank. There were 40 mice sacrificed in this experiment.

Therefore, we further transferred *Lat1*shRNA, pEGFP-N1 plasmid encoding *Lat1* (pEGFP-N1-*Lat1*) in ESCs and then induced decidualization. When cultured up to 72 h, we found that the transfection efficiency was about 40% by fluorescence microscope (Supplementary Materials Figure S4). Three sequences of *Lat1*shRNA were separately transferred in the decidualization of ESCs and then the efficiency of silence was evaluated by Western blotting. The results showed that the first sequence of *Lat1*shRNA (Si 1) was successfully silenced the expression of lat1 (Figure 3A, A1). In addition, pEGFP-N1-*Lat1* increased the expression of lat1 in the decidualization of ESCs (Figure 3C, C1). As expected, the raised expression of prl demonstrated the progression of decidualization, indicating that *Lat1*shRNA impaired decidualization of ESCs (Figure 3B, B1). In contrast, over-expression of lat1 by pEGFP-N1-*Lat1* plasmid enhanced decidualization progression by increasing prl expression (Figure 3D, D1).

**Figure 4:**
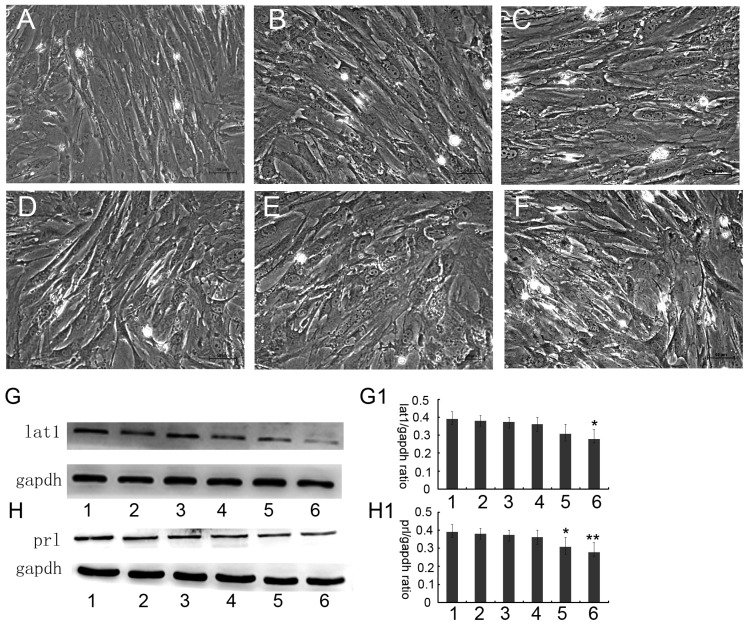
Effects of BCH on lat1 and prl expression in decidualization of ESC. Decidualizing ESC was treated with 0.05 μM, 0.1 μM, 0.5 μM, 2 μM, or 4 μM BCH separately were analyzed by Western blot. Morphology of ESC induced to decidualization for 72 h with different concentrates of BCH: (**A**) Blank; (**B**) 0.05 μM; (**C**) 0.1 μM; (**D**) 0.5 μM; (**E**) 2 Μm; and (**F**) 4 μM. GAPDH was used as a loading control. The expression of lat1 was decreased by 4 μM BCH treatment (**G**, **G1**). The protein levels of prl were similar trends to the lat1 protein levels (**H**, **H1**). Line 1–6: blank, 0.05 μM, 0.1 μM, 0.5 μM, 2 μM, and 4 μM BCH. * *p* < 0.05, as compared with blank. There were 40 mice sacrificed in this experiment.

Treatment of decidualization of ESCs with BCH, a competitive inhibitor of lat1transportation activity, was observed. Morphology ofdecidualization of ESC extracts was cultured up to 72 h at different concentrations and absence of BCH was shown in Figure 4A–F. BCH treatment also led to the down-regulation of lat1 protein and exhibited dose–effect relationship. The expression of lat1 could be significantly inhibited by 4 μM BCH treatment (Figure 4G, G1). We examined the effect of this treatment in the decidual progression. As shown in Figure 4H, H1, the decidualization of ESCs cultured in 4 μM BCH medium showed a lower level of prl expression.

### 3.3. Inhibitor of lat1 Transportation Activity Restrained Decidualization In Vivo

The decidua presented different morphological and functional areas: the antimesometrial decidua (AM) and mesometrial decidua (M) showed a clearly separated junctional zone (J). Tissues were collected on D8 from the pregnant mice treated with different concentrations of BCH. The 50 μg/mL and 5 μg/mL treatments in ISs produced a regression in the tissue with shrinkage of all decidual tissues, avoiding the differences between AM, M and J in 1 mol/L NH_4_OH treated ISs.

**Figure 5:**
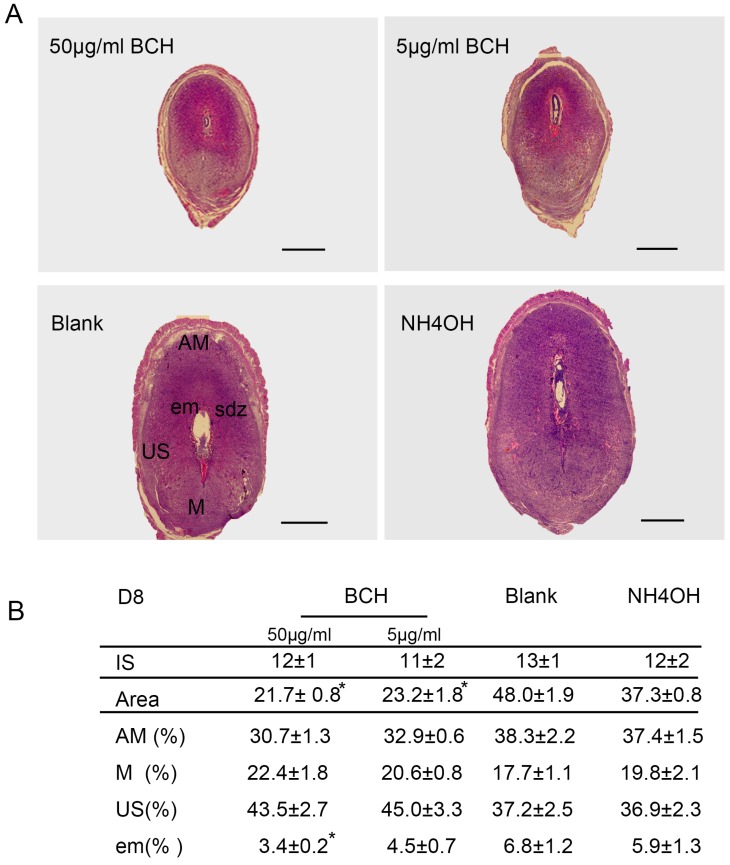
Morphology effects of BCH on D8 implantation sites after uterine horns injections on D4. (**A**) H&E staining on D8 from 50 μg/mL BCH, 5 μg/mL BCH, NH_4_OH (employed as a solvent) treated mouse, and normal pregnancy without any treatment; (**B**) Quantitative analysis. Table shows the number of implantation sites, total area of decidua and the percentage of tissue areas corresponding to the different regions within the ISs relative to the area of total decidua. Image J software analyzed the data that represented mean fold change ± SEM from at least 3 independent treatments.* *p* < 0.05. AM, antimesometrial decidua; M, mesometrial decidua; US, undifferentiated stroma; sdz, secondary decidual zone; em embryo IS, implantation sites. Bar = 75 μm. There were 15 mice sacrificed per group.

The 50 μg/mL and 5 μg/mL treatments in ISs showed residues of decidual tissues. The NH_4_OH treatment did not interfere with the decidualization process and resulted in normal ISs. These entire treatments did not have any impact on the IS and embryo resorption was not induced. All BCH-treated ISs developed decidual regions, AM, M and J, with a more compacted development of decidual area. However, though all decidual compartments were appropriately developed, the embryo was apparently smaller compared with NH_4_OH treatment and blank control groups (Figure 5). No change was observed in the ratio between AM and M to the complete decidua.

**Figure 6:**
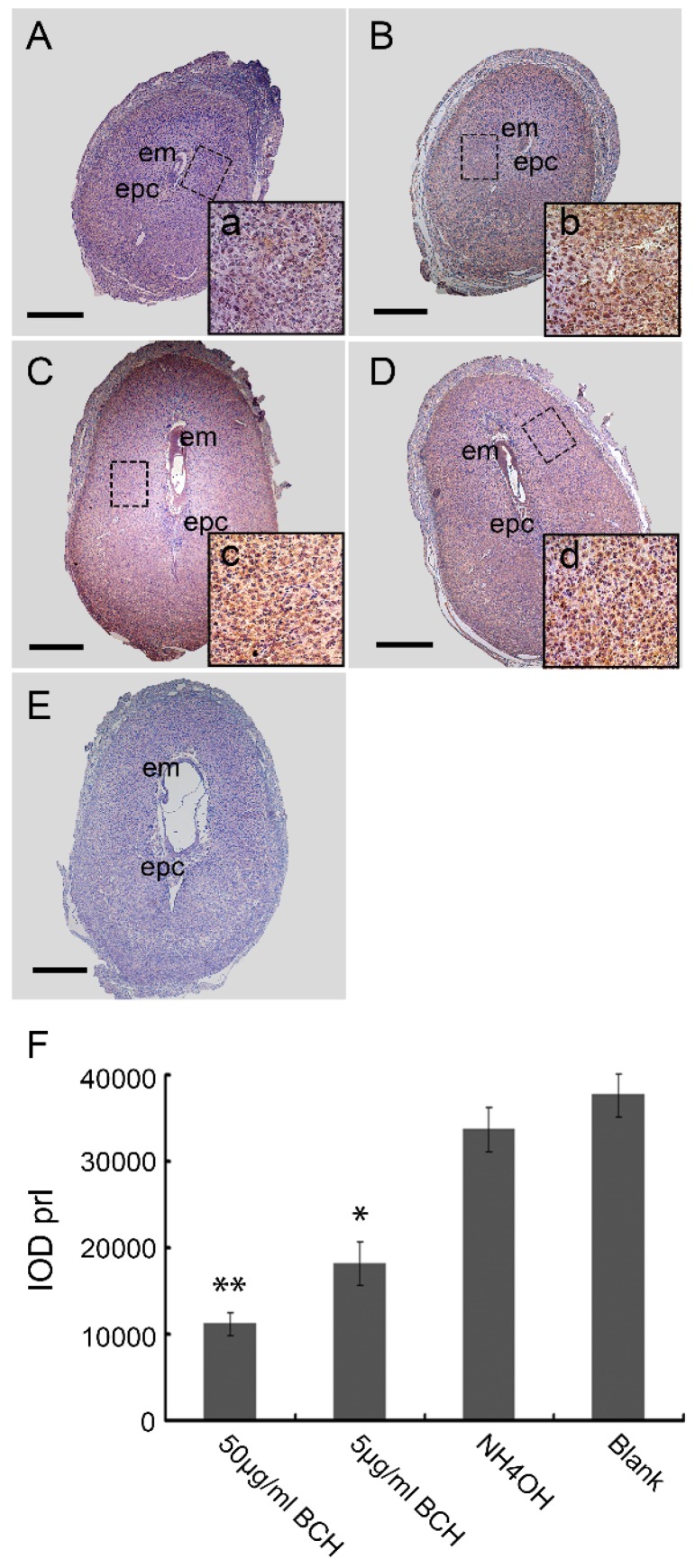
Localization analysis of BCH effects on prl expression on D8 of pregnancy. Original magnification immunostaining of prl on D8 of pregnancy, *40 times magnification*: (**A**) 50 μg/mL BCH; (**B**) 5 μg/mL BCH; (**C**) NH_4_OH; (**D**) Blank; (**E**) negative control; and (**F**) statistics of the prl positive expression in various groups was analyzed using the Image Pro-plus 6.0 software. The relative expression level was quantified using the IOD (integrated optical density), IOD = ∑ (optical density × area). a–d: inserts shows 400 times magnification. Bar = 75 μm. Decreased expression area was detected in the BCH treatment and exhibited dose–effect relationships (* *p* < 0.05; ** *p* < 0.01) as compared with blank control. There were 15 mice sacrificed per group.

**Figure 7:**
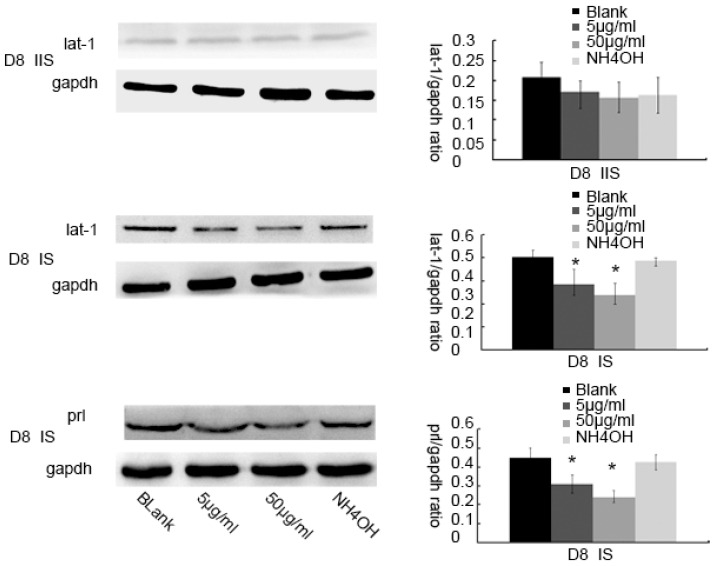
Quantitative expression of BCH effects on prl and lat1 expression on D8 of pregnancy. Samples from D8 of mouse with different treatments were distinguished between implantation sites and inter-implantation sites and then the tissues were analyzed for lat1 and decidualization marker prl protein expression. The expression of lat1 was decreased by BCH treatments in the implantation sites. The protein levels of prlshowedsimilar trends to the lat1 protein levels in implantation sites. These effects in implantation sites were not displayed in inter-implantation sites. IS: implantation sites, IIS: inter-implantation sites. * *p* < 0.05, as compared with blank control. There were 15 mice sacrificed per group.

To analyze in detail the decidualization process on BCH action, we studied lat1 and prl expressions, as shown in Figure 6. The relative expression levels of prl on D8 of pregnancy were decreased in the BCH-treated ISs which were counted by Image-Pro Plus (IPP6.0) software (Figure 6). In the case of BCH treated animals, 50 μg/mL BCH produced a remarkably reduced lat1 and prl expression (Figure 7). These results indicated that inhibitor of lat1 transportation activity of BCH restrained decidualization progression in vivo by down-regulating prl expression.

## 4. Discussion

In this study, we have investigated the role of lat1 transporter in mouse uteri in early pregnancy. Our data demonstrated that lat1 gene and protein expressions were upregulated from D5 to D8 of pregnancy in IS. It was previously reported that most of the transport systems are involved in the later stages of embryonic development, unlike these, lat1 was expressed in the preimplantation stages, which includes zygote, two-cell, four-cell, morula, blastocyst, and hatching blastocyst [2]. The development of uterine endometrium and embryo and changes in the maternal endometrium must be coordinated in the local environment. However, less is known about the effect of lat1 on maternal endometrial changes during implantation. Hence, in this study, we investigated the effect of lat1 on decidualization progression in vitro and in vivo and found that lat1 was mainly distributed in the luminal and glandular epithelium, ESC cytoplasm on D4 of pregnancy, increased expression in the decidualization of stromal cells in cytoplasm on D5 of pregnancy. At the IS from D6 to D8 of pregnancy, lat1 was located in the decidual cells cytoplasm and embryo. This observation was partly consistent with lat1 expression in the developmental embryo and provides more understanding in the synchronous changes of the uterus during embryo implantation.

Lat1 is a member of system l-type transporters also known as solute carrier family 7 (*Slc7a5*) or tumor associated gene-1 (*Ta1*). The *Lat1* gene is located on chromosome 16q24.3 and has high affinity for essential amino acids such as arginine, leucine, isoleucine, tryptophan, valine and participates in the transportation of neutral amino acids from extracellular to intracellular structures in a sodium-independent system [16,17]. Over expression of lat1 is a characteristic of many primary human cancers and may be related to tumor progression [18,19]. It has been demonstrated that higher lat1 expression was correlated with poor survival in human primary astrocytic tumors and glioblastoma multiforme [20]. In addition, the expression of lat1 was considered as one of the most significant predictors of outcomes, independent of all other variables. It is reported that high levels of lat1 are expressed in various tumors including mammary gland tumors, thyroid tumors, neuroendocrine tumors of the lung and hemangiopericytomas compared to normal tissues, and higher expression levels of lat1 in human colon cancers, breast cancers, head and neck cancers, genital cancers and soft-tissue sarcomas from patients were correlated with distant metastasis, suggesting a role for lat1 in the biological behavior of tumors [21,22,23]. Embryo implantation shared many similar features with tumor invasion. In morphology, decidualization of ESC with large and multi-nucleus can be seen as “tumor cells”. Trophoblast invasion is characterized by strict spatiotemporal regulation, which is mediated in an autocrine or paracrine manner by trophoblastic and uterine factors at the maternal-fetal interface [24]. Chrostowski et al. reported that on D8 of embryonic stage, both mRNA and protein expression of lat1 was restricted to trophoblast giant cells (TGSs) and played a key role in the mouse trophoblast cells Rs26 TS by invasion into mTOR pathway [25]. The mechanism of decidualization was associated with trophoblast invasion by forming primary decidual zone (PDZ) (morning of D6) and the secondary decidual zone (SDZ) (by D7), and this process was further accompanied with cell cycle balance. Furthermore, we have examined the effects of lat1 on decidualization progress in vitro and in vivo. Down regulation of lat1 expression by BCH significantly inhibited the expression of lat1 and prl by 5 μg/mL BCH, which suggested the negative effect of this treatment in decidualization progress. In contrast, lat1 activating experiments with pEGFP-N1 plasmid encoding lat1 showed an increased lat1 and prl expressions, demonstrating that lat1 has a positive effect in the decidualization progression in vitro. Thus, we speculated that lat1 might act as a dual regulatory function at the maternal-fetal interface in mouse embryo implantation.

BCH is a competitive inhibitor of Leucine transporter; this not only inhibits lat1 but also lat2, and showed substantial side effects when used clinically because lat2 is important for maintaining fundamental cellular activities in normal organs [26]. In rat C6 glioma model, BCH mediated the blockage of C6 glioma cells and increased the survival rate. These results suggest that lat1 could be one of the molecular targets in glioma therapy [27]. In primary human trophoblast cells and Rs26 TS cells, uptake of [^3^H] leucine was inhibited by BCH, which induced lat1 lower expression level [25,28]. However, in this study, we did not measure the effects of BCH on leucine uptake in the decidualization process of ESCs. However, we found a contrary phenomenon that 5 μg/mL BCH decreased lat1 expression in the decidualization process of ESCs. In our pervious study, Leucine addition showed no changes in mouse extravillous trophoblasts. Hence, further research is warranted to verify the existence unknown mechanisms if any.

IGF-1/AKT/mTOR signaling system has been identified as a nutrient regulator that specifically targets l-leucine [29]. mTOR signaling decreased the activity of human placental amino acid transporters and placental mTOR activity which is markedly decreased in human intrauterine growth restriction [30,31,32]. Amino acid signaling activates serine-threonine kinase mTOR, which then phosphorylates at least two proteins that are involved in the regulation of translation initiation, p70S6K and PHAS-I. Edinger et al. in his study noticed positive feedback amplification by over-expressing lat1 which is associated with the potential ability of FL5.12 cell proliferation between lat1 and mTOR [33]. Raptor- or rictor-silenced human primary trophoblast cells unaltered the expression of lat1 which indicated that mTOR signaling regulated trophoblast amino acid uptake by post-translational mechanisms, rather than by altering the expression levels. During embryo development from zygote to blastocyst and folliculogenesis in mouse, lat1 was confirmed as transporter of amino acids to regulate cell function and energy metabolism [34], but there were no evidences for the molecular mechanism. Decidualization is a dynamic balance that includes the process of proliferation and apoptosis and even seen as an energy supplier for embryo development before the formation of placenta. Therefore, further research on metabolism of amino acids in the process of decidualization associated with lat1 is needed.

Decidualization is affected by multiple factors including estradiol and progesterone [12]. Prl is present in the decidua and is induced by estradiol and progesterone through in vitro and in vivo decidualization of endometrial ESCs in rodents and humans [35]. Many hormones and growth factors showed a stimulatory effect on the transport activity of system L [36]. In this study, we chose prl as a decidual marker and also found that the lat1 protein expression was continuously increased in in vitro decidualization of ESCs induced by estradiol and progesterone up to 72 h. Our data demonstrated that lat1 might be a downstream molecule of ovarian steroid hormones and provided evidence for future investigations on the effect of hormones on system L transport and amino acid metabolism.

In view of the important role of lat1 in transportation of essential amino acids in the whole body, we chose to inject BCH through uterine horns as regional treatment on D4 of pregnancy. Fortunately, we established lat1 deficient mouse model which showed a decrease in the lat1 protein expression. To analyze further in detail the decidualization progression in vivo, we detected prl expression. All the treatments showed no interference with the IS but higher doses reduced the size of embryo and decidual area.

## 5. Conclusions

In conclusion, our study provided some lines of evidence that lat1 was actively involved in promoting stromal cell decidual progression both in vitro and in vivo through positive regulation of prl expression. Our study also described the role of lat1 in embryo implantation at the maternal–fetal interface and warrants further investigation regarding its pathophysiological role in human pregnancy.

## Supplementary Materials

The following are available online at http://www.mdpi.com/2072-6643/8/11/704/s1. Figure S1: Pregnant mouse uterus on Day 5 (D5): to identify the implantation and inner-implantation sites on D5, pregnant mouse uterus on D5 was injected with trypan blue. Figure S2: Morphology of ESC induced to decidualization for 72 h. Figure S3: Expression of prl in ESC after inducing decidualization by Western blot up to 72 h. Figure S4: Green fluorescent protein expression testifies the transfection efficiency in decidualization of ESC cells under a fluorescence microscope: (A) *Si-Lat1*; and (B) Over-Lat1. Bar = 100 um. Table S1: LAT-1-targeting shRNAs sequences.
