# Supplementary material for: Positive Regulation of Decidualization by l-Type Amino Acid Transporter 1 (lat1) in Pregnant Mice
Source: Nutrients. 2016 Nov 5;8(11):704. doi: 10.3390/nu8110704 (PMC5133091; doi:10.3390/nu8110704)
Supplement: Supplementary file 1 [file nutrients-08-00704-s001.docx]

Supplementary Materials: Positive Regulation of Decidualization by l-Type Amino Acid Transporter 1 (lat1) in Pregnant Mice

Xiaojie Wang, Dongmei Tan, Jing Ma, Hao Liang, Qian Zhang, Yi Tan, Jiang Wang
and Wenping Luo


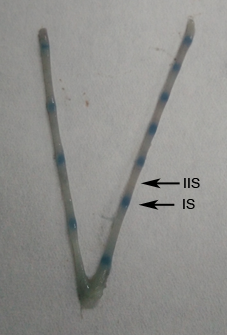


**Figure S1.** Pregnant mouse uterus on Day 5 (D5): to identify the implantation and inner-implantation sites on D5, pregnant mouse uterus on D5 was injected with trypan blue. The blue stained sections along the uterine horn show the implantation sites.


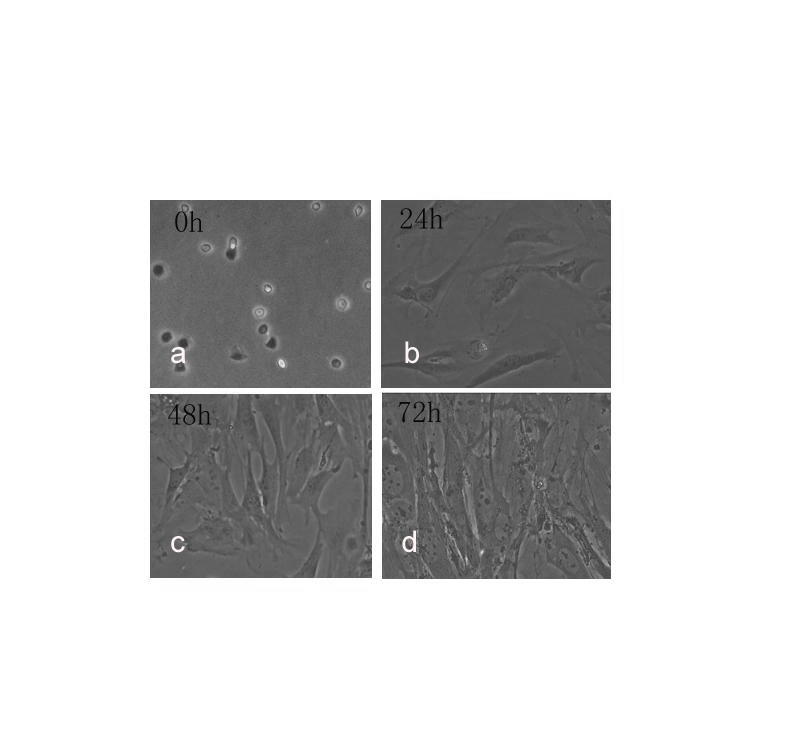


**Figure** **S2.** Morphology of ESC induced to decidualization for 72 h. After inducing decidualization with P4 and E2 treatments, cells were collected at 24 h intervals. Morphology verified that the decidualization induction was successful. (**a**–**d**) Morphology of ESC induced to undergo decidualization for 0 h, 24 h, 48 h and 72 h, respectively, at 200 times magnification.


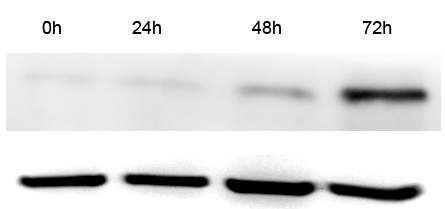


**Figure S3.** Expression of prl in ESC after inducing decidualization by Western blot up to 72 h. Decidualized ESC were collected at 24 h intervals. The expression of prolactin protein level was increased with the prolongation of time.


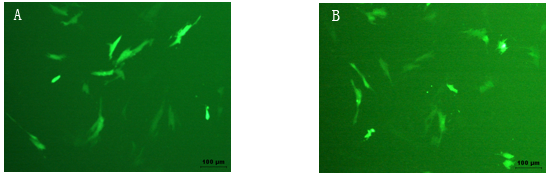


**Figure S4.** Green fluorescent protein expression testifies the transfection efficiency in decidualization of ESC cells under a fluorescence microscope: (**A**) *Si-Lat1*; and (**B**) Over-Lat1. Bar = 100 um.

**Table S1.** LAT-1-targeting shRNAs sequences.

| **ID** | **5′** | **Stem** | **Loop** | **Stem** | **3′** |
| --- | --- | --- | --- | --- | --- |
| LAT1-RNAi(1)-a | GATCCC | gcATTATACAGCGGCCTCTTT | CTCGAG | AAAGAGGCCGCTGTATAATGC | TTTTTGGAT |
| LAT1-RNAi(1)-b | AGCTATCCAAAAA | gcATTATACAGCGGCCTCTTT | CTCGAG | AAAGAGGCCGCTGTATAATGC | GG |
| LAT1-RNAi(2)-a | GATCCC | ctAGATCCCAACTTCTCATTT | CTCGAG | AAATGAGAAGTTGGGATCTAG | TTTTTGGAT |
| LAT1-RNAi(2)-b | AGCTATCCAAAAA | ctAGATCCCAACTTCTCATTT | CTCGAG | AAATGAGAAGTTGGGATCTAG | GG |
| LAT1-RNAi(3)-a | GATCCC | gaATTTCGTCACAGAGGAAAT | CTCGAG | ATTTCCTCTGTGACGAAATTC | TTTTTGGAT |
| LAT1-RNAi(3)-b | AGCTATCCAAAAA | gaATTTCGTCACAGAGGAAAT | CTCGAG | ATTTCCTCTGTGACGAAATTC | GG |
